# Expanding the Neurological Phenotype of Anderson–Fabry Disease: Proof of Concept for an Extrapyramidal Neurodegenerative Pattern and Comparison with Monogenic Vascular Parkinsonism

**DOI:** 10.3390/cells13131131

**Published:** 2024-06-29

**Authors:** Marialuisa Zedde, Ilaria Romani, Alessandra Scaravilli, Sirio Cocozza, Luigi Trojano, Michele Ragno, Nicola Rifino, Anna Bersano, Simonetta Gerevini, Leonardo Pantoni, Franco Valzania, Rosario Pascarella

**Affiliations:** 1 Neurology Unit, Stroke Unit, Azienda Unità Sanitaria Locale-IRCCS di Reggio Emilia, Viale Risorgimento 80, 42123 Reggio Emilia, Italy; valzania.franco@ausl.re.it; 2 Department of Neurosciences, Psychology, Pharmacology and Child Health, University of Florence, 50139 Firenze, Italy; ilaria.romani1@gmail.com; 3 Department of Advanced Biomedical Sciences, University of Naples “Federico II”, 80133 Napoli, Italy; alessandra.scaravilli@gmail.com (A.S.); sirio.cocozza@unina.it (S.C.); 4 Dipartimento di Psicologia, Università della Campania ‘Luigi Vanvitelli’, viale Ellittico 31, 81100 Caserta, Italy; luigi.trojano@unicampania.it; 5 Centro Medico Salute 23, Via O. Licini 5, 63066 Grottammare (AP), Italy; ragno.neuro@gmail.com; 6 Cerebrovascular Unit, Fondazione IRCCS Istituto Neurologico Carlo Besta, 20133 Milano, Italy; nicola.rifino@istituto-besta.it (N.R.); anna.bersano@istituto-besta.it (A.B.); 7 Head Diagnostic Dept and Neuroradiology Unit, ASST Papa Giovanni XXIII, 24127 Bergamo, Italy; sgerevini@asst-pg23.it; 8 Neuroscience Research Center, Department of Biomedical and Clinical Science, University of Milan, 20122 Milano, Italy; leonardo.pantoni@unimi.it; 9 Neuroradiology Unit, Azienda Unità Sanitaria Locale-IRCCS di Reggio Emilia, Viale Risorgimento 80, 42123 Reggio Emilia, Italy; pascarella.rosario@ausl.re.it

**Keywords:** Anderson–Fabry disease, MRI, advanced MRI, CADASIL, Parkinson’s disease, neurodegeneration, stroke, cerebrovascular disease, microbleeds, WMHs

## Abstract

Anderson–Fabry disease (AFD) is a genetic sphingolipidosis involving virtually the entire body. Among its manifestation, the involvement of the central and peripheral nervous system is frequent. In recent decades, it has become evident that, besides cerebrovascular damage, a pure neuronal phenotype of AFD exists in the central nervous system, which is supported by clinical, pathological, and neuroimaging data. This neurodegenerative phenotype is often clinically characterized by an extrapyramidal component similar to the one seen in prodromal Parkinson’s disease (PD). We analyzed the biological, clinical pathological, and neuroimaging data supporting this phenotype recently proposed in the literature. Moreover, we compared the neurodegenerative PD phenotype of AFD with a classical monogenic vascular disease responsible for vascular parkinsonism and cerebral autosomal dominant arteriopathy with subcortical infarcts and leukoencephalopathy (CADASIL). A substantial difference in the clinical and neuroimaging features of neurodegenerative and vascular parkinsonism phenotypes emerged, with AFD being potentially responsible for both forms of the extrapyramidal involvement, and CADASIL mainly associated with the vascular subtype. The available studies share some limitations regarding both patients’ information and neurological and genetic investigations. Further studies are needed to clarify the potential association between AFD and extrapyramidal manifestations.

## 1. Introduction

Anderson–Fabry disease (AFD) is a glycosphingolipidosis caused by a defect in the alpha-galactosidase gene [1,2], whose lower enzymatic activity determines the accumulation of glycosphingolipids in target cells, in turn causing their malfunction [3]. The cellular accumulation of glycosphingolipids can determine different dysfunctional phenotypes at the level of various organs and tissues; for example, at the cardiac level, the classic phenotype is represented by the occurrence of left ventricular hypertrophy [4]. AFD is a systemic disease, which affects every organ and tissue equally [4,5]. Its manifestations are therefore potentially polymorphic. The involvement of the central nervous system (CNS) includes different levels and mechanisms, from the vascular to the neuronal one [6]. In the field of neurological manifestations of the disease, elements are emerging in an increasingly coherent manner that support a neurodegenerative phenotype deriving from primitive neuronal involvement [7]. Indeed, recent reports suggest a potential association between AFD and Parkinson’s disease (PD), with observations of a higher prevalence of extrapyramidal signs and symptoms in a subset of AFD patients [8]. This association warrants further investigation, as its biological and pathophysiological rationales remain incompletely understood. Within this conceptual frame, a link has been recently proposed between AFD and an extrapyramidal clinical phenotype substantially indistinguishable from idiopathic PD [7], similar (although less characterized) to the association reported in Gaucher disease, another glycosphingolipidosis [9,10,11]. This neurodegenerative phenotype, which has conceptual, histopathological, neuroradiological, and clinical support, has been the subject of previous works by some authors [7], and it represent a different phenotype as compared with a classical example of monogenic vascular parkinsonism, “cerebral autosomal dominant arteriopathy with subcortical infarcts and leukoencephalopathy” (CADASIL). This review aims to describe the neurodegenerative phenotype of AFD and to highlight the differences in comparison with a pure vascular phenotype, represented by CADASIL. The neurological manifestations of AFD are part of a systemic disease context, so limiting the treatment to neurological manifestations does not mean that they are isolated. Indeed, in the vast majority of patients, multiple organs are involved.

## 2. Anderson–Fabry Disease: Clinical and Epidemiological Issues

### 2.1. Main Features from the Neurological Side

AFD is a hereditary sphingolipidosis resulting from a deficiency in the lysosomal enzyme alpha-galactosidase (α-GAL A), which is responsible for hydrolyzing terminal, non-reducing α-D-galactose residues in α-D-galactosides. This enzyme plays a crucial role in various catabolic processes, including the breakdown of glycoproteins, glycolipids, and polysaccharides. The α-GAL A enzyme is encoded by the GLA gene [1] (Xq22.1) [12]. AFD is an X-linked disorder affecting approximately 1 in 40,000 males. Unlike many other X-linked conditions, females carrying pathogenic GLA variants may exhibit systemic involvement and severity comparable to males. Precisely estimating the prevalence of AFD is challenging due to the heterogeneous effects of GLA pathogenic variants, which can lead to two distinct clinical phenotypes: “classic” AFD and “late-onset” AFD [13]. 

The deficiency of α-GAL A leads to the accumulation of globotriaosylceramide (Gb3) within lysosomes, particularly affecting endothelial cells, smooth muscle cells in vessel walls, cardiac muscle cells, endocardial cells, glomerular nephrons, neurons, and glial cells in the CNS, skin, and eyes. This widespread Gb3 buildup indicates systemic involvement in AFD, with organs such as the heart and kidneys being accessible for biopsy to confirm and evaluate Gb3 accumulation. However, CNS tissue biopsies are not typically part of the diagnostic process. Therefore, CNS involvement is primarily assessed through clinical events (e.g., transient ischemic attacks, cerebral hemorrhages, thrombosis, lacunar infarcts) and neuroimaging. CNS pathology in AFD is mainly considered a secondary effect of endothelial dysfunction, as evidenced by Gb3 accumulation in endothelial cells due to α-GAL A deficiency. Smooth muscle cells also play a role by releasing Gb3 and sphingosine-1-phosphate. However, neuropathological studies are limited, impeding a detailed understanding of the relationship between neurological symptoms and the underlying mechanisms of AFD. CNS involvement in AFD is relatively common, with a multispecialty screening study reporting its presence in 34% of patients [3]. The primary manifestation in the CNS includes vascular damage to the white matter and large vessels, such as vertebro-basilar dolichoectasia. There is limited information on direct neuronal and glial involvement in AFD. Traditional therapies, like enzyme replacement therapy (ERT), are ineffective in crossing the blood–brain barrier, complicating the treatment of neurological symptoms in AFD patients. Migalastat, a newer treatment option, has the potential to cross this barrier, but its direct effects on the CNS still require further investigation.

### 2.2. Biological Mechanisms 

The biological mechanisms of cell damage in AFD have not yet been fully elucidated in the peripheral and CNS. The accumulation of Gb3 and Lyso-Gb3 leads to cellular dysfunction, triggering a pathogenic pathway that progressively damages multiple organs, and in some cases, neurological manifestations might be the consequence of systemic involvement. In hemizygous AFD males, there is a significant accumulation of Gb3 in endothelial cells and Lyso-Gb3 in kidney cells (including smooth muscle cells and podocytes) and heart tissue (such as valves, cardiomyocytes, nerves, and coronary arteries). However, the precise role of Lyso-Gb3 analogues in AFD cellular pathology remains unclear. The molecular pathogenesis of AFD involves several pathological mechanisms, including mitochondrial and lysosomal dysfunction, GB3 accumulation, various Gb3 isoforms, globotriaosylsphingosine accumulation, endothelial dysfunction, and autophagy. 

Although peripheral nervous system complications are common in AFD patients and clinically well defined, their pathogenesis is still poorly understood. Most of the neurological symptoms can be attributed to neuropathy of small somatic and autonomic fibers or to the suffering of neurons from which they originate, but the mechanisms by which their damage develops are only partially known. In autoptic examinations of dorsal root ganglia, cytons are reduced in number, and the remaining cells are often balloon-shaped, with eccentric nuclei, and filled with granules reactive to lipophilic dyes [14,15,16,17,18]. Their perikarya are filled with granules and have few Nissl bodies. Similar findings are documented in sympathetic ganglia [18,19]; Auerbach’s and Meissner’s plexuses of the gastrointestinal tract; along with autonomic neurons of the spinal cord, brainstem, amygdala, and hypothalamus [20]. Electron microscopy shows electron-dense bodies that can be homogeneous or concentrically lamellated [14,15,16]. Similar inclusions are detected in endothelial and perithelial cells of ganglia capillaries and in fibroblasts, but not in satellite cells. Immunohistochemistry with monoclonal antibody to Gb3 confirms this selective pattern of neuronal involvement, albeit more extensively [18].

Studies performed on sensory nerve biopsies (especially the sural nerve) document a decrease in the number of myelinated fibers, often most evident for the thin myelinated Aδ fibers, and unmyelinated fibers [14,20,21,22,23], although unmyelinated fibers with diameters less than 0.5 µm usually appear to be increased in number, indicating axonal sprouting [22,24]. Lipid storage is almost never seen in axons and Schwann cells, which constantly show proliferation of their processes [14,20,21,22,23]. Targets of glycosphingolipid accumulation in peripheral nerves are the endothelial and perithelial cells of capillaries and the epineurial, perineurial, and endoneurial cells. The lumen of capillaries is usually narrowed but not occluded [14,20,21,22,23,24].

In skin specimens, a decrease in small fiber density and an abnormal morphology with more severe involvement of the distal leg than the proximal thigh (length-dependent small fiber neuropathy) is a well-documented finding [24,25,26,27]. Neuropathy involves both somatic epidermal and autonomic dermal nerve fibers. Also at this level, Gb3 deposits are seen in blood vessel walls and endothelial cells; sweat gland tubules; and perineurial, erector pilorum muscle, and dermal cells, but generally not inside the axons (which appear of altered morphology) or Schwann cells [24,27].

A number of hypotheses have been formulated to explain the selective neuronal involvement of AFD, but to date, data are inconclusive. The first hypothesis is of an endogenous, site-specific production of Gb3 and other stored glycosphingolipids [15,18,23]. However, such molecules are not normally detected in the brain, spinal cord, and dorsal ganglia of healthy adults [18]. Other hypotheses are that glycosphingolipids reaches neurons by retrograde axonal transport or are absorbed by cerebrospinal fluid or blood [18,24]. Dorsal root ganglia are situated outside of the blood–brain barrier, and dynamic-contrast-enhanced MRI studies can prove in vivo that, in healthy adults, the blood supply is higher in dorsal ganglia compared to spinal nerves (where perineural cells contribute to the blood–nerve barrier with tight junctions) [28,29,30]. In 11 male AFD patients with symptoms of peripheral nervous system involvement, this MRI technique documented severely enlarged ganglia, particularly those innervating the most distal dermatomes (i.e., L5, S1, and S2) with significantly decreased perfusion, while permeability between the vascular and the neuronal compartment of the proximal peripheral nerve appeared unchanged. Although less pronounced, similar results were found in 10 AFD females [31]. It could therefore be hypothesized that the Gb3 stored in sensory neurons does not originate in the ganglia capillaries or that it does so at a very early stage. Then, a progressive reduction in the permeability of the blood–ganglia barrier along with a hypoperfusion from nerve vessels would favor a degeneration of the nerve fibers with a length-dependent pattern (since the metabolic requirement of the longer fibers is greater) [31,32]. When the loss of nerve fibers exceeds a critical threshold, central pain conduction ceases, which could explain the spontaneous disappearance of painful acroparesthesias reported in the natural history of AFD [33]. This, however, should be accompanied by worsening deficits in thermal and noxious sensitivities [33].

Klein and co-workers [34] produced an in vitro model of neuronal disease using induced pluripotent stem cells derived from three Fabry patients and one control, aiming to better characterize the pathogenesis of small fiber neuropathy. Their model enables the study of disease mechanisms in a patient-specific manner. In their experiment, Gb3 accumulated ubiquitously in male-derived sensory neuronal somas and axons, and lysosomal integrity is impaired. Interestingly, AFD-derived neurons displayed a heat-induced Ca^2+^ increase; this finding may provide the basis for a molecular explanation of the episodic nature of AFD pain, along with the more often supposed vascular mechanism. In addition, specific attention has recently been paid to the consequences of Gb3 accumulation in skin keratinocytes and fibroblasts. These cells are key players in the processing of chemical and thermal stimuli and in cutaneous nociception, express pain-associated ion channels that are linked to the release of pro-inflammatory cytokines in patients with other small fiber neuropathies [35,36,37].

It has been documented in dermal fibroblasts of AFD patients that the accumulation of Gb3 can lead to a decrease in KCa1.1 channel activity and activate the Notch1 signaling pathway [38]. The resulting increase in pro-inflammatory cytokines (CCL2, TNF-β1, INF-γ) would sensitize nerve fibers in the immediate surroundings and thus contribute to the pain associated with AFD [38]. In another recent study, deposition of Gb3 in dermal fibroblasts was associated with an up-regulation of the cytokine-linked ion channel KCa3.1, which led to increased IL-8 production only in AFD females with a pain phenotype. IL-8 also led to sensitization of the nociceptor endings, reducing the induction thresholds for the action potential [39]. These studies support the possibility of an inflammatory contribution to the genesis of pain in AFD disease; in this regard, other authors hypothesized the involvement of another cytokine, TNFα, that shows higher values in the peripheral blood mononuclear cells of Fabry patients symptomatic for pain [40].

In the CNS, similar to what occurs in the peripheral nervous system, accumulation of glycosphingolipids is ubiquitous at the level of blood vessels (endothelial cells, smooth muscle cells, and pericytes) and more selective in some types of neurons. Monoclonal antibodies against Gb3 show that blood vessels throughout the brain and spinal cord are strongly immunoreactive, especially in the subpial vasculature [18]. Schibanoff [41] demonstrated in 1969 that the glycolipid concentration is higher in tissues rich in autonomic neurons than in the gray matter of the cerebral cortex. In brain autopsies [15,16,18,19,42], abnormal deposits are usually seen in neurons of the rostral hypothalamus, especially the supraoptic and paraventricular nuclei, the midline nucleus of the thalamus, the substriatal grey, the basal nucleus of the amygdala, the presubiculum of the hippocampus, the fifth and sixth cortical layers of the parahippocampus and the inferior temporal gyrus, the dorsal motor nucleus of the vagus, the superior and inferior salivatory nuclei, the Edinger–Westphal nucleus, the reticular formation of the pons and medulla oblongata, the trigeminal ganglia, and the substantia nigra (especially in non-pigmented neurons) [15,16,18,19]. Glia, particularly ependymal cells and subependymal astrocytes, and cells of the leptomeninges also contain abnormal deposits [15,18]. A mild astrocytosis and microglial proliferation in localized areas of the cerebral cortex and white matter is sometime observed, consistent with hypoxic-ischemic encephalopathy [18]. Overall, the autopsy data [43] provide valuable insights for establishing a solid foundation for pathophysiological hypotheses regarding the CNS involvement. These data help elucidate the putative biological mechanisms of damage. Autopsy studies have shown glycosphingolipid storage in muscle cells of cerebral vessels and neuronal tissue, particularly affecting the limbic system (e.g., basolateral nuclei of the amygdala, supraoptic, and paraventricular nuclei of the hypothalamus) and brainstem structures (e.g., substantia nigra, pontine reticular formation, dorsal efferent nucleus of the vagus, salivary nuclei, nucleus ambiguus, and mesencephalic nucleus of the fifth cranial nerve), as well as the spinal cord and peripheral nervous system. Despite the involvement of midbrain structures, notably the substantia nigra, extrapyramidal symptoms in AFD patients were only recently described. Zedde et al. [7] highlighted a model linking AFD and Parkinson’s disease (PD) based on glucocerebrosidase (GBA) deficiency observed in Gaucher disease [44]. GBA activity naturally declines with age [45], and GBA mutations can exacerbate this decline, leading to substrate accumulation and impaired α-synuclein trafficking. The connection between α-synuclein accumulation and lysosomes involves disruptions in the autophagy-lysosome system (ALS) and the ubiquitin-proteasome system (UPS), hindering the degradation of misfolded proteins and contributing to neurotoxicity and neurodegeneration in PD [46]. Additionally, ALS-related neuropathology and axonal degeneration have been observed in α-galactosidase A (α-GAL A)-deficient mice [47]. α-GAL A deficiency might reduce glucocerebroside levels, impacting the substrate available for GBA and suggesting similar pathological processes in both AFD and PD. Nelson et al. [48] found significantly reduced α-Gal A activity in the postmortem brains of ten advanced PD subjects. Another postmortem study [49] of two independent PD cohorts (*n* = 18 and *n* = 20) suggested an association between sporadic PD and decreased activity of several lysosomal hydrolases, including α-Gal A, in the substantia nigra. However, studies on lysosomal enzymes in cerebrospinal fluid (CSF) have not reported on GLA [50,51,52,53]. The most compelling hypothesis posits that GLA may have an independent role in PD, distinct from GBA, possibly through different pathways of brain damage resulting in unique phenotypes.

### 2.3. Neurological Involvement 

#### 2.3.1. Peripheral Nervous System

Symptoms related to peripheral nervous system involvement occur very early in the natural history of AFD, particularly in patients with the classic phenotype [54]. Neuropathic pain, of both somatic and visceral origin, and deficits in thermal and noxious sensitivities represent the most frequent clinical manifestations of its damage, along with dysautonomia [54,55]. Somatic pain typically manifests as acral-localized, symmetrical, burning pain with onset at the soles of the feet and subsequent spread to the palms of the hands and more proximal parts of the limbs (previously inaccurately referred to as acroparesthesia). Both evoked and spontaneous pain, which can be episodic or chronic in nature, have been described. Sudden changes in body (fever, exercise) or environmental (exposure to heat or cold, immersion in water) temperature usually act as triggers [56]. Some patients present with recurrent episodes of very intense, prolonged, agonizing pain, radiating proximally to the trunk and jaw. The intensity of these episodes (known as “Fabry crises”) is such that they lead the patients to the emergency room or confine them to bed. They may be accompanied by increased ESR and unexplained fever [57,58,59]. In most instances, pain begins during childhood or adolescence in both males and females. The Fabry registry and the Fabry Outcome Survey reported respectively that 60 to 80% of boys and 40 to 60% of girls are affected by neuropathic pain [60,61]. Later in life, this complaint may either spontaneously regress, persist, or become chronic. A first onset in adulthood is also possible [56]. In the same registries, the percentage of adults with pain was 62% to 81% of males and 41% to 65% of females, and the mean age of symptom onset was between 9 and 15 years in males and 13 and 20 years in females [62,63]. Pain and/or paresthesias are the most frequently reported symptom before age 5 years and develop also in children 2–4 years old [64]. In the Fabry Outcome Survey, pain became chronic in more than half of the males and nearly one-third of the females. Fabry crises accounted for 17% of pain syndromes in a German single-center cohort of 132 patients [56].

Visceral pain is another frequent and early manifestation of AFD. Patients report cramping discomfort generally localized to the mid-abdominal level [65]. The pain may be spontaneous or caused by meals and increased stress, and it may be associated with other gastrointestinal complaints such as diarrhea, constipation, nausea, and vomiting. Visceral pain is reported since a pediatric age. In the Fabry Outcome Survey, the overall prevalence of abdominal pain was 32.5%, with the same frequency among males and females, but with a higher rate in children than in adults [63]. More recently, 56% of males with a classic phenotype and 13% of males with a late-onset phenotype enrolled in the Fabry Registry were found to have visceral pain [66]. Although the etiopathogenesis of gastrointestinal symptoms is attributed to multiple factors (vasculopathy, neuropathy, dysmotility, dysbiosis, inflammation, malabsorption) [65,67], a recent cohort study hypothesized that pain is primarily a consequence of sensory neuropathy and visceral hypersensitivity [68].

Decreased thermal and noxious sensitivities are additional possible complications of AFD which should be investigated by specific neurologic tests (i.e., glass tubes filled with warm or cold water, Minnesota thermal disks, the “ice-bucket” test, or pinprick examination) because they may not be spontaneously reported by patients [55,69]. In addition, conduction is normal on electroneurographic tests because they only explore large-caliber fibers linked to extero-proprioceptive sensitivity and not to thermo-pain sensitivity. These manifestations are more frequent in males at the level of the feet and extend progressively in a centripetal direction [70]. Quantitative sensory testing and pain-related evoked potentials are used in neurophysiopathology laboratories to objectify these deficits, as nerve conduction studies are generally within the normal range in AFD patients [25,59,71,72]. The cold detection threshold appears to be more frequently impaired than the warm detection threshold (77% versus 23% of patients) [59]. Hypersensitivities to cold, warmth, and mechanical stimuli have also been documented with quantitative sensory testing [71,73]. Impaired sweating, gastrointestinal dysmotility, decreased saliva and tear production, impaired pupillary constriction, cardiovascular autonomic dysfunction, and orthostatic intolerance are the most reported symptoms of autonomic dysfunction in AFD patients. Abnormal sweating (usually hypohidrosis) has been reported at both distal and proximal body districts. This different topographic distribution with regard to pain and sensory symptoms is probably due to the involvement of sweat glands in addition to the autonomic nervous system, although a proximal, joint-level distribution is sometimes described for pain symptoms as well [55,70]. Hypo/anhidrosis causes a reduced tolerance to heat and physical exercise and a deficit in body thermoregulation, so AFD patients may develop episodes of fever of unknown origin complicated by the intense painful manifestations described above. The prevalence of the hypo/anhidrosis symptom among cohorts of adult patients ranges from 36 to 69% [27,73,74,75,76]. Episodes of unexplained hyperthermia seem to affect 20% of AFD patients at an early stage [73]. Along with abdominal pain, symptoms of bowel dysregulation (diarrhea, constipation, or both) are frequent gastrointestinal manifestations of AFD disease and are attributed to a deficit in bowel motility [77]. Diarrhea is the predominant complaint in males, constipation and bloating in females. Diarrhea is present in 26–57% of males with a classic phenotype and 12–39% of females, while constipation has a frequency of 9% in males and 17% in females [77,78]. Nausea was present in 12% of patients enrolled in the Fabry Outcome Survey, and there were minor differences between male and female patients. Using a wireless motility capsule to study the gut motility, delayed gastric, small bowel, or colon transit times have been found in 34% of Fabry patients, while abdominal pain was present in 81% of the same cohort [68]. This suggests that visceral pain has a complex etiopathogenesis that is not solely attributable to the autonomic intestinal transit dysfunction.

Impaired pupillary constriction with pilocarpine and reduced saliva and tear formation have been described in half of patients of a small AFD series. More recently, quantitative pupillary light reflex measurements were undertaken using an infrared dynamic pupillometry unit in a French cohort [79]. The amplitude and duration of pupil contraction and the latency of pupil dilation are reduced in patients with AFD compared to controls, suggesting a predominantly parasympathetic ocular dysfunction in AFD. A decreased heart rate variability was found in resting conditions (2-h ambulatory monitor) in pediatric males [80] and after standing up or forced breathing in adult populations. In the study by Biegstraaaten et al. [81], a heart rate variability reduction was reported in 17% of patients of both sexes undergoing autonomic cardiovascular function tests, but often in conjunction with an overt structural heart disease, suggesting that the cardiac autonomic neuropathy has only a partial pathogenetic role [81,82]. Pronounced orthostatic hypotension is rare even in more advanced stages of AFD, although in untreated male patients, standing up causes a significantly lower increase in blood pressure, particularly diastolic, than in healthy controls [83,84]. Erectile dysfunction, another typical symptom of other autonomic neuropathies, is also documented infrequently in AFD patients [81,82].

Symptoms of large nerve fiber impairment are less frequent and mostly related to nerve entrapment (e.g., carpal tunnel syndrome) or uremic neuropathy occurrence. Carpal tunnel syndrome occurred with a frequency of 10 to 27% in a small patient series and was due to the fibroblasts’ glycosphingolipid storage and increased extracellular matrix of the flexor retinaculum and connective tissue of the carpal canal [85].

#### 2.3.2. Central Nervous System

Stroke is the most frequently described clinical complication of CNS involvement in AFD, representing a major concern with regard to potential future disability [83,86]. Stroke occurred in 7% and 15% of males and in 4% and 11% of females of the large populations of the Fabry Registry and Fabry Outcome Survey [83,84], respectively. By including TIAs, the proportion of patients of both sexes with cerebrovascular events increased to around 25% in smaller cohorts [87,88]. The Fabry Registry shows that 46% of patients develop stroke before being diagnosed with AFD, and that in most cases, it occurs before overt renal or cardiac involvement. Most patients experience their first stroke between 20 and 50 years (median age: 39 years in males and 46 years in females), and 22% of them develop it before the age of 30 [83]. For all these reasons, many initiatives have been conducted to systematically screen for AFD in populations hospitalized for juvenile ischemic stroke [89,90,91,92,93,94,95,96,97,98,99,100,101,102,103,104,105,106,107,108,109]. A review of these works reports a prevalence of Fabry disease of 0.13% in males and 0.14% in females with stroke at a young age. This prevalence increases to 0.67% in males and 1.11% in females if GLA gene variants of uncertain or benign significance are included in the computing [110].

Ischemic stroke is the most common type of stroke in patients with AFD (83% of events). However, its pathogenesis has not been systematically studied [84]. Mechanisms postulated to be associated with ischemic stroke include dolichoectasia of the cerebral arteries, small vessel disease, cardiogenic embolism, impaired autonomic function, and alterations of blood components [111]. Dolichoectasia of intracranial vessels can lead to stroke through thrombosis of the main stem; artery-to-artery embolism; and stretching, distortion, and occlusion of its tributary vessels. This is most evident at the level of the basilar artery and progresses over time [112]. In 2009, Fellgiebel and colleagues [113] reported that the diameter of the basilar artery measured within the Time of Flight (TOF) MR angiography was better able to discriminate between Fabry patients and controls than diameters of other intracranial arteries (sensitivity: 95%, specificity: 83%). A similar result was more recently documented by Űçeyler and coworkers [114] in an independent series, but only in male patients. Other studies report that larger basilar artery diameters are associated with an increased risk of stroke in Fabry patients. Unfortunately, the association with the stroke location in the posterior cranial fossa has not been evaluated in these small cohorts [115,116]. In the Fabry Registry, the majority (70%) of strokes were related to small vessel disease, but this information was collected for only 39% of stroke patients [84]. The prevalence of small vessel disease (radiologically defined by the presence of lacunae, white matter hyperintensities, and/or microbleeds) was three times higher than that of clinically defined stroke (43% versus 14%) in another cohort of patients extrapolated from the Fabry Registry. Interestingly, in this study, the patients with residual stroke symptoms had the highest neuroimaging burden of small vessel disease, but the mechanism by which the stroke occurred was not always linked to small vessel occlusion, since non-lacunar infarcts were detected in their neuroimaging scans [117]. Data about the frequency of cardioembolism in AFD strokes are also scanty. Atrial fibrillation is a well-known complication in these patients, with longitudinal studies reporting an incidence between 3 and 31% (follow-up range 1.2–8 years) [118]. These figures may be influenced by the method chosen to identify atrial fibrillation, with 12-lead ECG and 24-h monitoring showing the lowest detection rates (2.9–10% per year) compared with the highest rates (19–31% per year) of continuous rhythm monitoring devices [119]. Since age is also associated with the occurrence of atrial fibrillation in AFD (OR 1.05–1.20 per 1-year increase in age), it is possible to hypothesize that cardioembolism is responsible for stroke in older AFD patients, where the burden of small vessel disease is also progressively higher [112]. Carotid intima-media thickness is another feature that has been frequently investigated, as in Fabry patients, it is considered a surrogate marker of the accumulation of glycosphingolipids in the vessel wall. Studies have detected a small but significant increase in the carotid intima-media thickness in AFD patients compared to controls, while carotid and femoral atherosclerotic plaques have been less represented (9.10% versus 43.94%; *p* < 0.001) [120,121,122,123]. The reason for this discrepancy is not clear; some authors hypothesized that the fibrotic tissue reaction triggered by the Gb3 accumulation may create a barrier to the penetration of cholesterol and the formation of plaques [123], but a specific contribution could be made by the careful control of vascular risk factors to which these patients are subjected in their reference centers. Impaired autoregulation of cerebral vessels is hypothesized to contribute to the stroke pathogenesis in addition to the mechanisms described above. Brachial flow-mediated dilatation is reduced in Fabry patients compared to healthy controls [122,123]. This suggests an inefficiency of peripheral vasoregulatory mechanisms. At the CNS level, nuclear medicine techniques have documented that neuronal hypometabolism exists in relatively hyperperfused (with increased cerebral blood flow) brain areas [124,125,126]. This is particularly evident in those regions where white matter hyperintensities are detected on brain MRI and may suggest a primary cerebrovascular abnormality. Other authors have used Transcranial Doppler to document reduced cerebral vasoreactivity to CO_2_ and to blood pressure oscillations, although not all data have been consistent [127,128,129]. Impaired neurovascular coupling (reduced increase in cerebral blood flow velocity in the posterior cerebral artery) in response to visual stimulation has also been documented [121,125]. Finally, an increase in the expression of adhesion molecules at the level of endothelial cells, the release of inflammatory cytokines, and endothelium-derived microparticles cause a pro-thrombotic imbalance in patients with Fabry disease that may favor stroke occurrence [122,123].

Cerebral hemorrhage is a less frequent and later cerebrovascular complication than ischemic stroke. The median age of hemorrhagic stroke patients in the Fabry Registry is 48 years (26 to 57) in men and 58 years (33 to 65) in women [84]. This is about 10 years older than for ischemic stroke. Degeneration of the cerebral small vessels secondary to deposition of glycosphingolipids and aging, in addition to hypertension and antiplatelet/anticoagulant drugs, are considered to be contributing factors for cerebral hemorrhage in AFD [130,131,132]. Cerebral microbleeds on MRI sequences are described in 11% to 30% of Fabry patients. They seem to be associated with male sex, concurrent white matter hyperintensities, and chronic kidney disease [132].

Aseptic meningitis with recurrent ischemic strokes and macrovascular stenoses is a rare and serious neurological complication of Fabry disease. Among 107 French patients with FD followed between January 1995 and September 2023, its prevalence was 3.7% [133]. Montardi and colleagues [133] recently reviewed this atypical manifestation of Fabry disease, identifying 29 cases. Patients presented with acute or chronic meningeal symptoms (particularly headache) and elevated levels of C-reactive protein. Focal neurological deficits may be reported up to the time of onset. Fabry aseptic meningitis occurs at a young age: the median age of patients was 29 years (IQR 26–41), and 59% were males. Meningitis is often lymphocytic (65%), and cerebrospinal fluid pleocytosis is usually mild. Stroke was detected in 57% of patients, and in 62% of cases, there was a cerebrovascular recurrence at follow-up. Large artery stenosis was present in 17% of patients. The pathophysiology is unknown; the main hypothesis is that the Gb3 accumulation within the pia and arachnoid membranes may induces a lymphocytic influx or trigger an inappropriate autoinflammatory reaction.

## 3. Neurodegeneration in Anderson–Fabry Disease

### 3.1. A Clinical Point of View

Neuronal involvement in AFD was postulated in early neuropathological studies, and a “neurodegenerative” clinical and cognitive phenotype has increasingly been described with supporting evidence. This phenotype often presents motor symptoms with extrapyramidal, Parkinson’s-like features. Assessing neurological involvement in AFD typically relies on neuropathological examination, though obtaining brain tissue for diagnostic purposes is crucial. This is especially important for diseases like AFD, which do not usually require neuropathological analyses for diagnosis. Some isolated investigations of non-autopsy brain tissue in AFD patients have been reported, often in cases where patients underwent brain biopsy or neurosurgical procedures for other conditions. Additionally, limited published data from autopsy series of AFD patients [43,134,135] mostly occurred before ERT and lack the electron microscopy typically used for diagnosing AFD in other tissues (e.g., kidney, heart, and skin). One particularly interesting finding from autopsy studies [43] is the documented storage of glycosphingolipids in muscle cells of cerebral vessels of various sizes, neuronal tissue, and specific brain structures. These include components of the limbic system (such as the basolateral nuclei of the amygdala and the supraoptic and paraventricular nuclei of the hypothalamus) and brainstem structures (such as the substantia nigra, pontine reticular formation, dorsal efferent nucleus of the vagus, salivary nuclei, nucleus ambiguus, and mesencephalic nucleus of the fifth cranial nerve) as well as the spinal cord and peripheral nervous system. Despite the documented involvement of midbrain structures, including the substantia nigra, extrapyramidal clinical manifestations in AFD patients had not been described until recently. This absence of previous descriptions might be due to the complex phenotype of AFD and the significant changes in its natural history following the introduction of ERT. Before the advent of enzyme replacement therapy (ERT), cardiac and renal issues were the primary factors reducing life expectancy in classic AFD patients, with CNS involvement arising partly from systemic damage. However, with improved management of cardiac and renal conditions post-ERT and increased life expectancy, the burden of CNS damage secondary to systemic disease has diminished. Conversely, the identification of late-onset variants has facilitated the recognition of milder phenotypes, including neurological aspects. The association between AFD and PD was first highlighted over 20 years ago through the publication of two cases: a 68-year-old man and a 57-year-old woman, both presenting with a DOPA-responsive clinical extrapyramidal phenotype [136,137]. The man [136], who was autoptically confirmed to have AFD, experienced atypical parkinsonism beginning at age 63, characterized by axial signs (gait and postural instability), mild symmetrical rigidity, and pyramidal signs (mild right hemiparesis, generalized hyperreflexia, and positive Babinski sign on both sides). Brain MRI revealed multiple T2 hyperintensities in the basal ganglia and deep white matter regions. No information was available about his response to levodopa. The woman [137] was diagnosed through molecular and genetic testing, identifying the R301P GLA mutation. She developed mild parkinsonism at age 46, with motor complications including axial signs (gait impairment with freezing) and a good response to levodopa. Her brain MRI showed extensive leukoencephalopathy with multifocal ischemic lesions, including in the head of the right caudate nucleus. Lohle et al. [8] found that individuals with causal GLA mutations exhibit a clinical bradykinetic motor phenotype, including slower gait and reduced hand speed, but without the classic prodromal features of PD (hyposmia/anosmia, autonomic dysfunction, depression, and REM sleep behavior disorder). This differentiates them from individuals with GBA mutations [138]. Recently, a survey [139] of AFD patients with known GLA mutations reported personal and familial prevalence of PD. This survey, distributed through the National Fabry Disease Foundation and the Fabry Support and Information Group in the United States, used a validated questionnaire [140] to ascertain family history of PD and focused on previously diagnosed PD and its treatment in participants [141,142]. Participants also reported if any first-degree relatives showed common PD symptoms (resting tremor, shuffling gait, stooped posture, decreased arm swing, and rigidity). This approach mirrored methods used to determine the penetrance of monogenic PD forms due to LRRK2 and GBA mutations [141,142]. Among 90 genetically confirmed AFD patients, 2 reported a prior PD diagnosis (2/90, 2.2%), both over 60 years old. The prevalence of PD diagnosis among participants over 60 was 8.3% (2/24). A Kaplan–Meier survival analysis indicated an 11.1% age-specific risk of PD by age 70. The two AFD/PD patients carried the p.Y134X mutation and the p.E59V variant. The p.Y134X mutation is associated with a classical AFD phenotype [139,143,144], while the p.E59V variant was previously unreported, although a missense mutation at the same GLA gene position (E59K) [143] is causative of classical AFD, and in silico models suggest the damaging potential of the variant [145]. Regarding family history, 4 out of 81 families (4.9%) had a first-degree family member with a prior PD diagnosis. This study had limitations, including self-reporting and lack of direct patient evaluation and follow-up, but it suggests an association warranting further analysis, particularly in late-onset AFD phenotypes due to specific GLA mutations.

### 3.2. Neurodegeneration in Anderson Fabry Disease: A Neuroradiological Point of View

#### 3.2.1. Conventional MRI Markers

**Figure 1:**
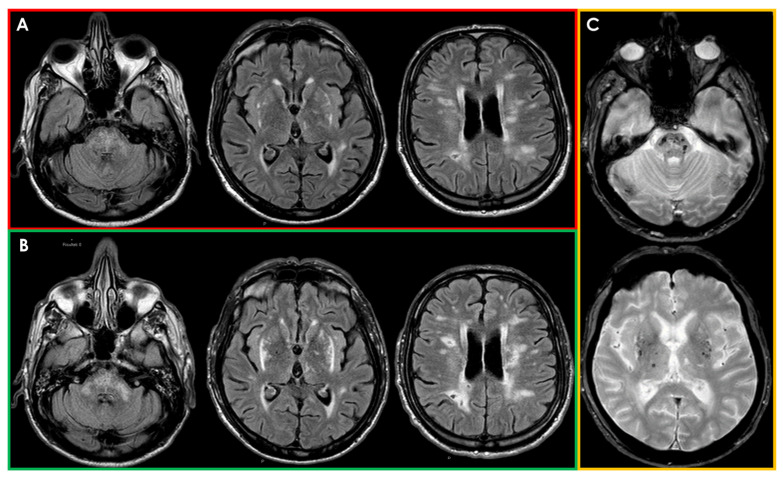
MRI of a patient with “classical” AFD. Panels (**A**,**B**) show axial FLAIR sequences highlighting the WMHs and lacunar burden at the baseline and after 1 year, respectively. In Panel (**C**), T2* sequence points out to the microbleeds’ burden in the pons and in the basal ganglia and external capsule.

Supporting the hypothesis of primary neurological involvement in AFD, several studies employing longitudinal neuroimaging and neuropsychological assessments suggest a possible neurodegenerative pathogenesis independent of cerebrovascular disease. For instance, a study by Lelieveld et al. [146] examined neuropsychiatric symptoms and brain structural alterations in 14 AFD patients over an 8-year follow-up period. The study revealed a progressive decrease in hippocampal volume, despite no observed cognitive changes. This hippocampal volume loss, which was not significantly correlated with changes in white matter hyperintensities (WMHs), suggests pure neuronal involvement and a neurodegenerative phenotype in AFD patients. MRI findings of hippocampal atrophy align with autopsy studies [18,147,148] demonstrating Gb3 storage in selective cortical and brainstem areas, including hippocampal neurons and ganglion cells, particularly in the presubiculum and parahippocampal gyrus. This Gb3 storage may induce cellular damage through oxidative stress and compromised energy metabolism, potentially leading to cell death. Furthermore, the lack of correlation between WMH volume and hippocampal atrophy in AFD further supports the hypothesis of pure neuronal involvement in the disease. It appears that hippocampal involvement may serve as a hallmark of neuronal damage in AFD, albeit without overt clinical manifestations beyond depression. Besides these neurodegenerative MRI markers, it is possible to identify the classic signal alterations expression of cerebrovascular lesion typical of AFD, as previously indicated and summarized in Figure 1. 

#### 3.2.2. Advanced MRI Techniques

Along with conventional MRI [149], which represents the gold standard imaging technique to assess brain involvement in AFD for diagnostic purposes, in recent years, advanced imaging techniques have expanded the knowledge about the pathophysiological mechanisms underlying CNS impairment, both in symptomatic and asymptomatic subjects, providing volumetric, structural, functional, and metabolic information. The application of advanced imaging techniques in AFD, as voxel-based morphometry (VBM), has shed light on brain volume changes and expression of tissue loss due to neurodegeneration, allowing for a quantitative, accurate, and reproducible assessment, especially in patients without severe cerebrovascular disease [150]. A global volume loss seems to occur sporadically and in more advanced phases of brain involvement, especially in the presence of significant WMH load or, as expected, in patients with brain infarcts (territorial or lacunar) [149,151,152]. In general, no differences in terms of regional gray matter (GM) volume loss were found in AFD patients compared to healthy controls [150,153,154,155], except for a cluster of reduced GM density at the level of the thalami [156]. Hippocampal atrophy has also been reported [146,149]. Furthermore, a harmonious reduction of intracranial tissue volumes was reported in AFD, suggesting a possible abnormality of neural development in this condition [156].

**Figure 2:**
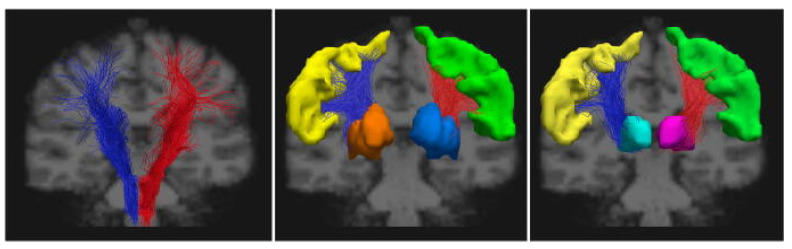
Reconstructed tracts in a 29-year-old female healthy control. Reconstructions of the cortico-spinal, the cortico-striatal, and thalamo-cortical tracts are showed from the left to right (left tracts in red, right tracts in blue), with the green and yellow areas indicating the left and right precentral gyri, respectively. Finally, the dark blue (middle image) and purple regions (on the right) of interests represent the left striatum (as the sum of the caudate nucleus and putamen) and the thalamus, while orange (middle image) and light blue (on the right) indicate the contralateral regions. Reprinted from [163].

On the other hand, via the application of diffusion tensor imaging (DTI) models (Figure 2), a sensitive technique able to quantitatively detect brain tissue microstructural changes not visible on conventional MRI [157], widespread WM damage has been reported in AFD [158], mainly expressed by an increase in global WM mean diffusivity (MD) [113,153,159], reduced fractional anisotropy (FA) values throughout the brain [150,160], or a combination of both [161], suggesting increased water content and loss of myelin integrity and axons. This extensive microstructural involvement was also confirmed in the absence of WM lesions and extending beyond WMH [161] in normal-appearing WM (NAWM) at the level of the frontal, parietal, and temporal lobe [150,161], thus interpreted as a biomarker of microvascular injury at early stages [158]. Furthermore, microstructural involvement was also found in the thalamus and in periventricular regions [159]. This phenomenon was explained as possibly due to microvascular modifications [162] and shows strong correlations with cognition and disease severity [161].

Although DTI studies suggest widespread WM microstructural damage, some reports have suggested a preferential involvement of posterior brain territories in terms of hyperperfusion or metabolic disturbance [124,164], coupled with a higher lesion probability in posterior periventricular regions [161], suggesting that microstructural damage occurs independently of these factors.

**Figure 3:**
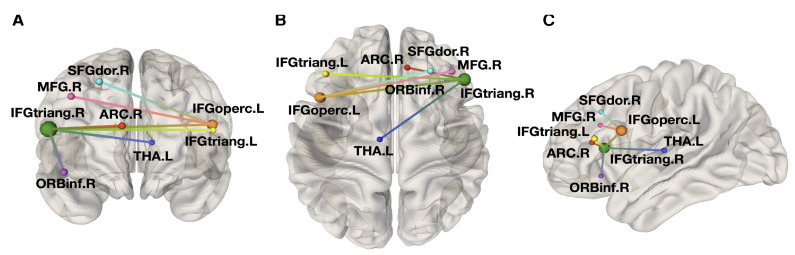
Network-based statistics results from 10.1093/braincomms/fcac187. Image shows coronal (**A**), axial (**B**), and sagittal (**C**) views of the subnetwork with decreased structural connectivity in Fabry disease patients compared with HCs emerging from a network-based statistics analysis with a primary threshold of t = 3.0. Its eight nodes, whose sizes reflect the number of connections in the subnetwork (i.e., node’s degree), are the left thalamus (THA.L), right inferior frontal gyrus—opercular part (IFGoperc.L), left inferior frontal gyrus—triangular part (IFGtriang.L), right anterior cingulate and paracingulate gyri (ACG.R), right superior frontal gyrus (SFGdor.R), right middle frontal gyrus (MFG.R), right inferior frontal gyrus—triangular part (IFGtriang.R), and right inferior frontal gyrus—orbital part (ORBinf.R). Reprinted from [160].

Similarly, a few functional MRI (fMRI) studies have confirmed the occurrence of significant functional changes in AFD, with widespread structural disconnection and functional reorganization, independent from major cerebrovascular events [153,160,164], affecting motor and cognitive functions, the former expressed by increased activation of additional cortical regions during motor tasks [165] and alteration of the cortico-striatal pathway [164], with the latter explored via resting state (RS) fMRI technique, demonstrating changes in the default mode network (DMN) [153]. See also Figure 3.

Other advanced MRI techniques have also been applied in AFD. Among these, interesting results emerged in studies using the Magnetization Transfer Ratio (MTR) and quantitative MRI (qMRI), which showed a decreased myelin density and bound pool fraction in NAWM [152,166], increased susceptibility values in striatonigral pathway [167,168], and no significant changes in the pulvinar [169]. Very recently, the application of machine learning methods (such as the brain-age paradigm, a sensitive index of structural brain health) have also led to the suggestion of accelerated cerebral aging in this condition [170]. 

As confirmed by the abovementioned advanced imaging studies, although AFD has long been considered a condition in which neurological involvement was related only to the presence of major cerebrovascular events, prodromes of neurodegeneration are present in this condition, with particular reference to motor functions, mainly expressed as motor slowing, postural instability, rigidity, and tremor [8], suggesting a possible link between AFD and PD [139,171,172]. Investigation on motor impairment in AFD has demonstrated the presence of functional alteration of the extrapyramidal system, with major alteration of the corticostriatal pathway in RS-fMRI [150], increased susceptibility in the striatum, coupled with a volumetric reduction in substantia nigra (SN) via quantitative susceptibility mapping (QSM) [168], suggesting iron accumulation in the striatonigral system, similarly to that present in Parkinson’s disease [173] and atypical parkinsonisms [174]. This involvement of SN was also pathologically confirmed in FD in terms of severe neuronal loss, along with moderate Gb3 and severe Lewy bodies deposition [175].

## 4. Monogenic Vascular Parkinsonism: The Example of CADASIL

### 4.1. Clinical Issues

CADASIL is a monogenic autosomal dominant arteriopathy, and it is the most common heritable cause of stroke in adults [176]. CADASIL is caused by cysteine-altering missense mutations in one of the 34 epidermal growth-factor-like repeat (EGFr) extracellular domains of the NOTCH3 protein. NOTCH3 is expressed only to the vascular smooth muscle cells in normal human adult tissues, but its exact function remains unknown [176]. CADASIL is considered the most prevalent monogenic cerebral SVD, with an estimated prevalence of 2–4:100,000, which varies widely geographically [177]. However, recent large-scale genomic studies have revealed a higher prevalence (1:300) of pathogenic NOTCH3 variants among the general population, resulting in the assumption that this disease is currently underdiagnosed [177].

CADASIL has a highly variable severity, even within the same family, ranging from a severe early-onset to a much milder late-onset presentation. The strongest known CADASIL modifier is the NOTCH3 variant position. In 2023, Hack et al. identified three clinically distinct EGFr risk categories, classified as either low (LR-EGFr), medium (MR-EGFr), or high risk (HR-EGFr) [177]. These risk categories are used to predict disease severity in individuals with CADASIL, from a classical severe CADASIL phenotype to very mild subclinical phenotypes. Significant differences were reported between different EGFr risk categories in the most important clinical and neuroimaging outcomes.

Clinically, CADASIL is characterized by five main symptoms: migraine with aura (MA), subcortical ischemic events, mood disorders, apathy, and cognitive impairment. Among these symptoms, MA is reported in 20% to 40% of patients and is often the first manifestation of the disease [176]. Visual or sensory aura symptoms usually last 20–30 min and are followed by a headache lasting a few hours. By contrast, migraine without aura has the same frequency in patients with CADASIL and the general population [176]. 

Despite the high incidence of MA, its mechanism of action is still unknown. Blood vessels may play an important role in the migraine attacks, as animal studies show a strong vascular component in the pathophysiology of MA [178]. On the other hand, in CADASIL, vasoreactivity of small penetrating arteries is likely to be compromised due to vascular fibrosis and the degeneration of smooth muscle cells [179], and this could be related to the pathogenesis of migraines. Severe depressive episodes are generally present in 20% of patients, and decreased voluntary behavior associated with the absence of motivation has been detected in about 40% of CADASIL patients, independently from depression [176]. Executive dysfunction is usually detected with a specific test and is commonly associated with alterations in memory and attention; onset is at 35–50 years of age [176]. Cognitive impairment worsens with strokes and increases the risk of death. Brain atrophy is usually seen, and its extent is related to cognitive and disability scales [176]. 

Subcortical ischemic strokes are the most common clinical manifestation and can be the first to occur. In 60–85% of symptomatic individuals, transient ischemic attacks and strokes occur at a mean age at onset of 49 years (range from 20 to 70 years of age) [176]. Ischemic episodes typically present as lacunar syndrome and are usually subcortical. In most cases, conventional vascular risk factors are not present. Strokes due to large vessel occlusion have occasionally been reported, but they are not typical [176]. Intracerebral hemorrhage (ICH) was considered rare in CADASIL and related to hypertension or antithrombotic treatment. However, an increased rate of ICHs in CADASIL was recently described, mostly located in the thalamus and lobar regions [180,181]. The risk of ICH appears to be independently related to the presence of cortical microbleeds in the brainstem or a count > 10 [180]. The relationship between genotype and risk of ICH is uncertain. In fact, some studies have warned of an increased risk of ICH linked to genetic mutations, such as R544C [182]. Recurrent strokes over several years may progressively lead to severe leukoencephalopathy, which may rarely be responsible for parkinsonism due to potential damage to the substantia nigra, putamen, caudate nucleus, and basal ganglia-thalamocortical circuit [173]. In particular, in CADASIL, there is some evidence of selective involvement of the arterioles of the lenticular nucleus due to degeneration of vascular smooth muscle cells, loss of autoregulation, and fibrosis [183]. Pseudobulbar palsy or a clinical picture compatible with progressive supranuclear palsy (PSP) has been reported in some cases [176,182]. However, other cerebrovascular disorders, such as Binswanger’s disease or subcortical gliosis, have also been described to be associated with this parkinsonism [182]. The 123I-FP-CIT SPECT in patients with CADASIL and parkinsonism showed symmetrical or asymmetrical reduction of tracer uptake in the putamen, with inconstant caudate involvement [184].

However, no evidence of significant associations between PD and NOTCH3 gene mutations has been found so far [185]. All patients with CADASIL and asymmetric parkinsonism presented a limited response to levodopa, probably due to vascular involvement of the putamen.

### 4.2. Cognitive Issues

Vascular cognitive impairment (VCI) and dementia are the second most frequent clinical manifestations of CADASIL. The earliest sign in most cases is impairment in executive function and processing speed [176,186,187], but all cognitive domains, including memory, can be involved [184,188,189,190,191]. VCI is progressive and isolated in up to 10% of patients and progresses with age (especially after the age of 60 years) to a picture of severe dementia, in most cases associated with motor impairment, gait disturbances, and, later, pseudobulbar palsy [176]. Usually, in early stages, cognitive processing speed decreases, and even patients without global cognitive impairment experience a slowing in accomplishing neuropsychological tasks [188,192]. Later, the cognitive slowdown increases, and variable and progressive impairments on tests tapping attention and executive functions can be detected. Eventually, further cognitive functions are involved, and the patients gradually lose their functional autonomy [176,193].

However, disease course and severity show a high interindividual variability, even within the same family [176]. The reasons for such heterogenous phenotypes are still uncertain. Some studies pointed out that additional vascular risk factors [194,195] and specific mutation sites [195] might be associated with a more rapid progression rate and severity. Stroke and male sex are two known risk factors for VCI and dementia [194,195], whereas no strong association between epidermal growth factor-like repeats and VCI has been ascertained [191,194]. Several studies [191,196,197,198] showed a strong association between the presence and number of subcortical lacunes with VCI and degree of cognitive impairment. Beyond the load of lacunar lesions, however, it would be important to understand the role of cerebral localization of these lesions in determining cognitive status in CADASIL patients. This appears particularly relevant, as it has been shown that lacunar lesions can be associated with secondary demyelination, thus contributing to development of cortical atrophy [199,200].

Nonetheless, the role of apparently “asymptomatic” lacunar lesions in precipitating VCI in patients with CADASIL should be clarified to define therapeutic guidelines. Indeed, a recent Cochrane review [201] did not identify evidence that antithrombotic therapy can yield any clinically relevant cognitive benefit and/or improvement in performance in neuropsychological tests or in daily activities in people with cerebral SVD without dementia.

Another source of heterogeneity across studies could be represented by the methods used for detecting VCI in patients with SVD. Some authors employed neuropsychological tests focusing on executive function and processing speed, such as the Brief Memory and Executive Test [202], whereas others adopted more global cognitive assessment tools such as the Montreal Cognitive Assessment [203]. In a recent study [204], we demonstrated that, even in the lack of clinically relevant general cognitive deterioration, constructional abilities are often impaired in genetically confirmed CADASIL; such disorders appear to be mainly related to impairments of control executive functions and to the reduction of grey matter volume in the parietal and frontal brain regions, even if CADASIL is a predominantly subcortical vascular disease. The above considerations underline the need for a wide-range neuropsychological assessment to detect VCI in CADASIL and to comprehend the relationships between cognitive defects and brain lesions. Studying asymptomatic individuals with CADASIL offers a unique genetic model to understand preclinical VCI and dementia, as CADASlL is a pure model of vascular dementia with younger onset with respect to the more frequent neurodegenerative diseases, and its cognitive profile is similar to that of sporadic vascular dementia due to SVD. In patients with CADASIL, progressive vascular lesions lead to a sort of secondary cognitive frailty [205,206], which in its turn contributes to development of a real frailty syndrome in association with ageing. Recent data suggest that an age-related decline in Notch3 signaling in both murine and human brain vessels can trigger a progressive decline in vascular function and culminate in neurodegeneration [207]. On this basis, it has also been suggested that aging-related Notch3 deficiency in patients with CADASIL might underpin small vessel disease-related neurodegeneration [207]. 

Assessing asymptomatic individuals over time could help to comprehend whether VCI and dementia in CADASIL can be better explained by accumulating brain lesions, in a sort of a diffuse smoldering pathological process that may affect the entire brain, including the cerebral cortex. Testing this hypothesis could open novel therapeutic approaches, as at the moment, no disease-modifying treatment is available. The identification of a cholinergic deficit in CADASIL [208,209] resulted in a placebo-controlled 18-week randomized clinical trial with donepezil in a sample of symptomatic but nondemented patients (mean age 54.8 years) [210]. This study did not show any significant effect on global cognitive efficiency or daily activities but produced some differences in secondary endpoints concerning executive function. However, the relatively small number of patients and the brief follow-up period would suggest promoting further longitudinal studies on larger samples recruited at early disease stage and follow-up for longer time periods.

### 4.3. Neuroimaging Issues

The vascular lesion responsible for CADASIL is characterized by a unique type of arteriopathy that is neither arteriosclerotic nor amyloid. It predominantly affects small perforating cerebral arteries, where the primary pathological feature is the accumulation of granular and osmiophilic materials within the vascular smooth muscle cell (VSMC) membranes [211,212]. This initial accumulation leads to thickening and expansion of the extracellular matrix, which can also be observed, albeit to a lesser extent, in extracerebral arteries such as skin arterioles [213]. Over time, VSMCs degenerate and are replaced by fibrotic tissue within the vessel walls, causing stenosis in the distal segments of medullary arteries. As a result, cerebral vessels lose their capacity for autoregulation and become dependent on blood pressure for proper function [214]. These pathological changes in small vessels ultimately result in small infarcts within the white matter, deep gray matter, and pons.

Neuroimaging findings are a cornerstone for CADASIL diagnosis, and they rely mainly on conventional MRI, which offers higher sensitivity and specificity compared to CT. The characteristic MRI findings cover the whole spectrum of SVD markers [215], in particular WMHs; lacunar infarcts, mainly located in the centrum semiovale, thalamus, basal ganglia, and pons; and cerebral microbleeds. These findings might be identified in both symptomatic and asymptomatic patients with a confirmed NOTCH3 mutation [216]. Usually, years before the onset of the first clinical manifestations, brain MRI discloses WMHs, sometimes in the typical locations (anterior temporal poles and external capsule) [176]. The exact age at earliest onset of first MRI abnormalities is uncertain, but they become constant by around 35 years in all patients [196]. The full neuroradiological spectrum of CADASIL includes the evidence of diffuse, symmetrical, and often progressive WMHs, detected on T2-weighted/fluid-attenuated inversion recovery MRI; multiple lacunar infarcts; dilated perivascular spaces; cerebral microbleeds on T2* gradient recall echo or susceptibility-weighted imaging; and brain atrophy on T1-weighted MRI. Although no pathognomonic neuroradiological pattern can be identified for CADASIL, the presence of WMHs on MRI at the level of the anterior temporal poles, frontal lobes (sub-insular areas and superior frontal gyrus), and external capsule bilaterally is a characteristic feature of CADASIL, which could therefore guide clinicians in the diagnostic pathway [176,217,218].

In fact, the predominant location of hyperintensities in the temporal lobes, the symmetrical distribution, and the frequent involvement of the external capsule should be helpful in differentiating CADASIL from other disorders involving the white matter. The abnormalities first appear as punctiform or nodular but later become more diffuse and symmetrical. Lacunar infarcts appear later in life and occur usually in the same areas. It is not uncommon to find small areas of increased signal on DWI-MRI, suggestive of recent infarcts, often asymptomatic [215]. Finally, microbleeds are present on gradient echo images in about 25–69% of cases, predominating in the basal ganglia [193,219]. As previously pointed out, clinical and radiological manifestations appear at different ages but are typically observed in most patients over 35 years old [176]. Age correlates positively with the prevalence and severity of MRI-detected brain changes, which can appear 10–15 years before clinical symptoms manifest. Asymptomatic individuals show less severe and less diffuse MRI signal abnormalities compared to symptomatic patients, indicating a link between these abnormalities and symptom severity [220].

In more detail, T2-weighted MRI images, which are more sensitive than T1-weighted images, often detect early CADASIL manifestations as areas of increased signal intensity, particularly in the periventricular white matter [176]. Studies comparing MRI findings in NOTCH3 mutation carriers to age-matched non-carriers have reported WMHs predominantly in the periventricular region, anterior temporal pole, external capsule, and frontal and parietal areas. These lesions are typically symmetrical. Less affected regions include the posterior temporal and occipital lobes, basal ganglia, thalamus, pons, and internal capsule. Arcuate fibers are generally spared. Corpus callosum involvement, rare in sporadic SVD, has been noted in some CADASIL cases. Higher frequencies of dilated perivascular spaces (PVS) have also been observed in CADASIL patients [221,222]. Most structural MRI studies on CADASIL patients have revealed diffuse and regional brain atrophy [221]. Diffusion tensor imaging (DTI), which assesses white matter microstructural integrity, has shown significant diffusion changes in CADASIL patients, indicating widespread microstructural damage. The basal nuclei and thalamus are particularly affected [221,223].

**Figure 4:**
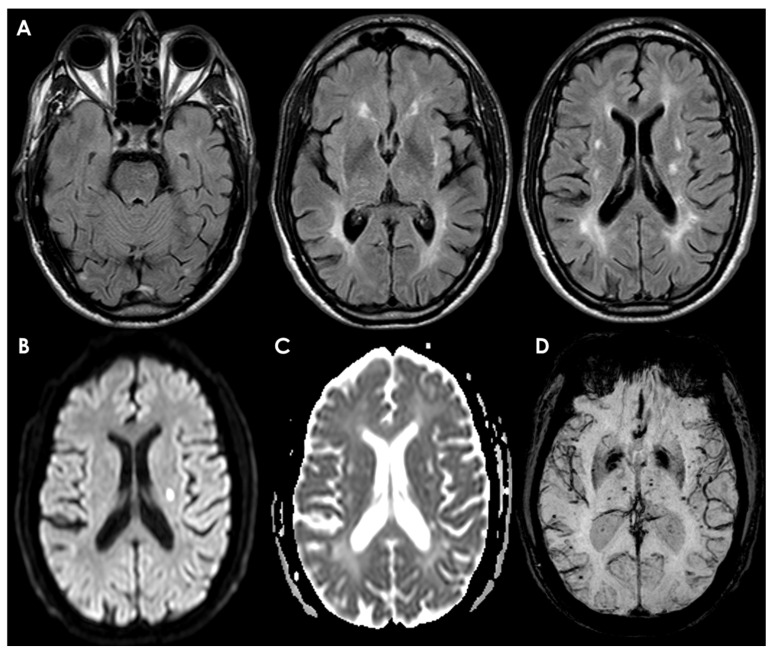
Brain MRI of a patient genetically diagnosed as having CADASIL. In panel (**A**), the axial FLAIR slices show only mildest temporal WMHs and bilateral involvement of the external capsule with old and recent subcortical infarctions. The recent one is in the left corona radiate, and it is hyperintense in DWI-MRI (**B**) and hypointense on the ADC map (**C**). The burden of deep and lobar cerebral MBS is illustrated in panel (**D**).

For differential diagnosis, MRI markers specific to CADASIL are crucial [224]. Anterior temporal lobe involvement on MRI is highly specific for CADASIL, with some studies reporting a sensitivity and specificity up to 90% and 100%, respectively [176,225]. However, these features are sometimes seen in individuals without NOTCH3 mutations and may be absent in early disease stages. Lesions in the external capsule have also been proposed as diagnostic features. MRI differences between CADASIL patients and non-carriers are not consistently found across studies. Temporal pole WMHs, while indicative of CADASIL, are not exclusive to it and may not always be present. The variability in MRI presentations and the influence of different genotypes further complicate diagnosis. For instance, anterior temporal involvement is less common in patients with cysteine-sparing NOTCH3 mutations [226]. Overall, CADASIL cannot be reliably differentiated from other SVD forms based solely on MRI. While certain MRI features like anterior temporal pole and external capsule involvement suggest CADASIL, additional diagnostic information is essential (see Figure 4).

Studies investigating the relationships between neuropathology and MRI markers provide crucial insights into the pathological processes underlying MRI signal abnormalities in CADASIL. These studies offer a more comprehensive interpretation of MRI findings, although systematic investigations in CADASIL patients remain limited. Dichgans et al. [227] attempted to correlate in-vivo MRI with autopsy findings. MRI scans were performed on 16 CADASIL patients, while autopsies were conducted on seven different patients. However, due to the lack of available MRI data for the autopsied cases, the study was mainly observational and did not directly link imaging to pathological variables. Autopsy findings revealed lacunes and diffuse white matter changes due to demyelination, axonal loss, gliosis, and extracellular space enlargement. Focal accumulations of hemosiderin-containing macrophages were noted in six of the seven brains. The authors hypothesized that homogeneous rounded foci of signal loss on T2*-weighted images likely corresponded to hemosiderin deposits from cerebral microbleeds, though other causes could not be excluded due to methodological limitations. Viswanathan et al. [228] studied neuronal apoptosis in four CADASIL patients who had MRI scans performed within a year prior to death. Apoptotic neurons were found in all cases at autopsy, particularly in cortical layers 3 and 5, correlating with the extent of subcortical WMH and axonal damage. The patient with more severe apoptosis had greater volumes of WMH and lacunes, suggesting a contribution to cerebral atrophy in CADASIL. Jouvent et al. [229] conducted a combined postmortem neuropathological examination and high-resolution 7-T MRI (HR-MRI) on a 53-year-old CADASIL patient. They found that linear hypointensities on T2* images, representing microvessels, and two subtypes of intracortical infarcts were visible. These findings indicate that high-resolution MRI can detect intracortical infarcts, which might be missed with lower resolution imaging. Liem et al. [230] investigated iron deposition using high-resolution 7-T MRI and histopathological examination in three CADASIL patients. Iron deposits were found in the caudate nucleus, putamen, and globus pallidus, corresponding to signal loss on T2*-weighted MR images. This suggests that MRI hypointensity is linked to progressive iron accumulation. Yamamoto et al. [211] used digital images of serial in-vitro slices from a CADASIL patient’s temporal pole to simulate MR images, proposing that MRI hyperintensities in the temporal pole reflect enlarged perivascular spaces, myelin depletion, and axonal damage. Indirect measures in other studies have shown associations between serum neurofilament light chain, a marker for axonal damage, and MRI markers like brain volume, WMH, lacunes, and CMBs. High-resolution 7-T MR technologies have also been used to estimate pathological processes in CADASIL. For instance, Liem et al. [231] found that luminal diameters of lenticulostriate arteries were unaffected, suggesting lacunes in the basal ganglia were not due to artery narrowing. De Guio et al. [232] demonstrated reduced venous density in white matter using HR 7-T MRI, while Fang et al. correlated retinal vessel changes with 7-T MRI markers, linking CM or small infarcts to retinal vessel integrity. Overall, these studies underscore the importance and validity of MRI in detecting pathological alterations in CADASIL. Future systematic studies combining MRI and post-mortem examinations could optimize MRI sequences for detecting and classifying brain lesions in vivo, enhancing our understanding of CADASIL’s neuropathology.

## 5. Conclusions

The link between monogenic vasculopathies and PD is made up of multiple elements and mechanisms, which refer to the different characterizations of the disease in question. AFD has a connotation with cerebral vascular damage, mainly in the context of SVD, but also a component of neuronal damage, direct and not mediated by endothelial dysfunction and other vascular mechanisms, which accounts for a neurodegenerative clinical phenotype, including extrapyramidal. This hypothesis is also supported by neuroradiological studies, both conventional and advanced. In contrast, CADASIL provides a model for an almost pure cerebral vascular disease, and the phenotypic consequences are those of extrapyramidal syndrome secondary to vascular encephalopathy, the expression of which is in fact much more marked than in AFD. The two diseases both have atrophy among the neuroradiological markers, but with a different characterization, i.e., degenerative in AFD and vascular in CADASIL. 

Knowing these aspects and the underlying cellular and molecular mechanisms can help with the correct classification, including clinical, of the patient, and with the definition of a pathophysiology of brain damage, for which there are several questions still unanswered.

## Data Availability

Not applicable.
